# Seed germination of seven desert plants and implications for vegetation restoration

**DOI:** 10.1093/aobpla/plw031

**Published:** 2016-07-11

**Authors:** Liming Lai, Lijun Chen, Lianhe Jiang, Jihua Zhou, Yuanrun Zheng, Hideyuki Shimizu

**Affiliations:** ^1^Key Laboratory of Resource Plants, Beijing Botanical Garden, West China Subalpine Botanical Garden, Institute of Botany, Chinese Academy of Sciences, Xiangshan, Beijing 100093, China; ^2^Institute of Applied Ecology, Chinese Academy of Sciences, Wenhua Road, Shenyang 110016, China; ^3^National Institute for Environmental Studies, Tsukuba 305-8506, Japan

**Keywords:** Germination, Horqin sandy land, light, Psammophyte, restoration, temperature

## Abstract

Germination cues reflect the conditions under which a species is likely to succeed in recruitment. Therefore, knowledge of the seed germination characteristics of key plant species in desertified areas is essential for restoration. The aims of this study were to evaluate the seed germination responses of seven native species, and to explore the implications for vegetation restoration. Seeds of seven desert species were sown in Petri dishes and subjected to various temperature and light conditions. The seeds germinated well at day/night temperatures of 25/15 °C and 30/20 °C but poorly at 35/25 °C. Seeds germinated best in the dark, and final germination percentages of all species were strongly inhibited at a photon irradiance of 1000 µmol m^−2^ s^−1^. Based on these results and the environmental conditions of their natural habitat, *Agropyron cristatum* and *Artemisia halodendron* are best adapted to shifting sand dunes: *Elymus dahuricus*, *Caragana korshinskii* and *C. microphylla* for semi-fixed sand dunes: and *Medicago sativa* and *Melilotus suaveolen* for fixed sand dunes. If seeds are sown in early May, they will likely be buried in sand, and the precipitation and temperature conditions will be suitable for seedling survival.

## Abstract (precis)

In this paper, we evaluated the seed germination responses of seven desert species to temperature and light and explored the implications for vegetation restoration. Both temperature and photon irradiance influenced seed germination of these species. Based on these results and the environmental conditions of their natural habitat, suggestions about plant choices for different habitats including shifting sand dunes, semi-fixed sand dunes and fixed sand dunes are given. Based on the precipitation and temperature conditions, early May is suitable for seed sowing in the study area.

## Introduction

Desertification resulting from human activities has become a global environmental issue and a serious socioeconomic problem (D’Odorico *et al.* 2013). Although combating desertification has been focused and carried out over several decades, the situation is getting worse, especially in arid and semi-arid areas, where it exacerbates the local problems of poverty and poor environmental quality (Su and Zhao 2003; Wang 2003; Reynolds *et al.* 2007). Vegetation rehabilitation is considered to be an effective way to combat desertification and restore ecosystems (Li *et al.* 2004). Because native plants are highly adaptable to their local environment, establishment of new populations of native plants is one of the main strategies used in restoration (Abella *et al.* 2012). However, the natural restoration process is still slow in arid regions. Plants have adaptations that promote survival and growth in different areas (Bellard *et al.* 2012). The success of establishment greatly depends on seed germination, since this process determines when and where seedling growth begins (Tobe *et al.* 2005). Seed germination is the first crucial growth stage, and its adaptation to different environmental conditions affects the survival of individual plants and community dynamics (Dürr *et al.* 2015). For successful seedling establishment, seeds should not germinate when there is a high risk of drought, extremes of temperature or adverse light conditions for growth. Thus, germination cues reflect the climatic conditions under which a given species is most likely to succeed in recruitment (Brändle *et al.* 2003).

During the early stages of succession, adaptation of seed germination to the local environment can help invading species to colonize new habitats (Halpern *et al.* 1997). Seeds have special germination mechanisms that allow them to adapt to different environments (Baskin and Baskin 2014). In extreme desert conditions, seeds do not germinate until the water supply is sufficient for seedling survival (Gutterman 1972). In saline environments, seeds of halophytes germinate faster than those of glycophytes, and they maintain viability even under extreme saline or osmotic stress to recover and germinate when the water potential increases (Ungar 1995). In wetlands in temperate regions, seeds of aquatic plants do not germinate until the water recedes; therefore, the non-flooded period coincides with the growing season (Baskin and Baskin 2014).

The environmental control of seed germination is a complex process. Angevine and Chabot (1979) suggested that seed germination under natural conditions means the individual has ‘bet its life’ on the favourability of environmental conditions for seedling establishment. Consequently, favourable environmental selection mechanisms are closely related to plant establishment. Among the factors that can affect seed germination, temperature and light are two of the most important. Temperature is the main factor regulating dormancy in temperate regions (Baskin and Baskin 1988). It has a dual effect on seed germination and regulates seed dormancy and germination percentages and rates when temperatures are within the range of those favourable for germination temperatures (Fenner and Thompson 2005). Seed germination occurs between minimum and ceiling threshold temperatures, and the highest germination percentage is at the optimal temperature (Dürr *et al.* 2015). Thus, characterization of the threshold values for germination can define the limits of the thermal environment that a species will tolerate, as described recently for wild grapevine (*Vitis*) seeds (Orrù *et al.* 2012). Knowledge of these threshold values is useful for determining the timing of seed sowing for vegetation restoration (Baskin and Baskin 2014; Dürr *et al.* 2015).

For many plants that inhabit sandy soils, light is one of the most important signals for germination (Gutterman 1993). For example, seeds of the sandy-habitat species *Trachyandra divaricata* cannot germinate in red light (Bell 1993), and germination of the two grass species *Festuca hallii* and *Koeleria macrantha* is strongly inhibited by light (Federico *et al.* 2014). Seeds of the sandy-land species *Artemisia ordosica* and *Artemisia sphaerocephala* only germinate in darkness (Zheng *et al.*, 2005*a*,*b*; Lai *et al.* 2010). In sandy areas, seeds are often buried in sand at various depths, or they may disperse into shaded areas (Maun 1998; Zheng *et al.* 2005*c*). Thus, seeds may germinate under a certain range of light intensities. Since seed germination determines when and where seedling growth begins, the success of seedling establishment depends greatly on seed germination responses to the environment (Tobe *et al.* 2005). Therefore, to develop effective restoration strategies, it is important to study the effects of different light intensities on the seed germination responses of plant species used in restoration programmes.

Vegetation restoration can be viewed as a succession series (Zhang *et al.* 2005; Zheng *et al.* 2006). At each stage of the restoration process, there is a dominant species with particular life history traits that contribute to its dominance (Zhang *et al.* 2005). Several native species are widely used for vegetation restoration in the Horqin Sandy Land in north China; *Agropyron cristatum*, *Artemisia halodendron*, *Elymus dahuricus*, *Caragana korshinskii*, *C.* *microphylla*, *Medicago sativa* and *Melilotus suaveolens*. These plants grow in a complicated landscape that includes moving, semi-fixed and stabilized sand dunes. Therefore, the germination characteristics of these plant species should be evaluated to determine that are most appropriate for use in restoration of various kinds of sandy land. For example, pioneer species used to restore vegetation on moving sand dunes should be able to germinate in the dark or under low light intensities. Once established, these species could create a suitable environment for the establishment of other plant species.

The aim of the present study was to evaluate the effects of various temperatures and levels of photon irradiance on seed germination of seven plant species that are native to the Horqin Sandy Land in northeast China. The results of this study will contribute to a better understanding of the recruitment and performance of these species, and they will allow better selection of species for use in vegetation restoration. We aimed to answer the following questions: (i) what temperatures are suitable for seed germination of each species? (ii) How does the germination pattern vary under different photon irradiance? (iii) If there are differences in seed germination, how are they related to species selected for restoration of different kinds of sandy lands?

## Methods

### Study sites

The Horqin Sandy Land is located in the semi-arid zone of northeast China. At the southwestern end of the Horqin Sandy Land (42°55′N, 120°41′E), annual mean precipitation is 366 mm, with 70–80 % of the precipitation occurring between May and September. Annual mean evaporation is around 1935 mm, and annual mean temperature is 6.8 °C (Naiman Desertification Research Station, Chinese Academy of Sciences). The soils are classified as Cambic Arenosols and are susceptible to wind erosion (Zhao *et al.* 2007).

### Seed collection and storage

Ripe seeds of seven species (*A. cristatum*, *Artemisia* *halodendron*, *E. dahuricus*, *C. korshinskii*, *C. microphylla*, *M. sativa* and *Melilotus* *suaveolens*) were collected from plants in the Horqin Sandy Land in 2004 (Table 1). For every species, a set of 20 square plots, 100 m^2^ per plot, were set up, total sampled area was 18 000 m^2^. Seeds were collected from plants in each plot to obtain an adequate representation of genetic diversity. After seeds were cleaned and air dried, they were stored in cloth bags at 4 °C until used.

**Table 1.** Characteristics of seven plant species included in this study.

Species name	Family	Ability to fix nitrogen	Life form	Regeneration	Seed collection	Store period (months)
*Agropyron cristatum*	*Gramineae*	No	Perennial, grass	Seeds	September	7
*Artemisia halodendron*	*Compositae*	No	Perennial, shrub	Seeds	October	7
*Elinelymus dahuricus*	*Gramineae*	No	Perennial, grass	Seeds	September	9
*Caragana korshinskii*	*Leguminosae*	Yes	Perennial, shrub	Seeds	September	9
*C. microphylla*	*Leguminosae*	Yes	Perennial, shrub	Seeds	September	9
*Medicago sativa*	*Leguminosae*	Yes	Perennial, grass	Seeds	October	12
*Melilotus suaveolens*	*Leguminosae*	Yes	Biennial, grass	Seeds	October	12

### Germination experiments

Germination experiments were carried out in temperature-, humidity- and light-controlled growth chambers (KG-306SHL-D, Koito Co., Ltd., Tokyo, Japan) and were started on April 2005. Seeds were not scarified before used. Seeds were kept in darkness or under a 14-h light/10-h dark photoperiod, with light supplied by cool white fluorescent lamps.

Seeds were sterilized with ultraviolet radiation for 10 min and then placed on 3-fold Toyo No. 1 filter paper (Toyo Roshi Kaisha Ltd., Tokyo, Japan) in Petri dishes (90 mm diameter × 15 mm depth). Distilled water was added until the seeds floated but were not inundated. Each treatment had 5 replicates of 25 seeds. Seeds were inspected daily under dim fluorescent light (10 µmol m^−^^2^ s^−^^1^). Emergence of the radicle was the criterion for germination (Baskin and Baskin 2014). Germinated seeds were discarded after counting. Experiments were terminated after 30 days (Zheng *et al.* 2005*b*).

### Effects of different temperature regimes on germination

To test the effects of temperature on germination, seeds were kept in the dark under 4 day/night temperature regimes: 20/10 °C, 25/15 °C, 30/20 °C and 35/25 °C. Relative humidity of the chamber was set to 70 %.

### Effects of photon irradiance on seed germination

The day/night temperature and day/night relative humidity were set to 25/15 °C and 60:50 %, respectively. Seed germination was evaluated under five different levels of photon irradiance: darkness (0), 15, 62.5, 250 and 1000 μmol m^−^^2^ s^−^^1^. Seeds were incubated in clear plastic boxes covered with layers of white and black plastic netting to achieve the different photon irradiances, or in black wooden boxes made with two layers spaced 4-cm apart. In each layer, there was a long, narrow aperture and the aperture in one layer was offset from that in the other layer. This construction allowed air to flow freely into and out of the box but prevented entry of light.

### Statistical analysis

Germination was measured using two indices: final germination percentage (FGP) and germination rate (GR). The FGP is the percentage of seeds sown that germinated. The GR (speed) was estimated using a modified Rozema index of germination rate (Rozema 1975): Σ(100*G_i_*/*nt_i_*), where *n* is the number of seeds in each treatment (25 each replicate) and *G_i_* is the number of seeds germinated on day *t_i_*(*t_i _*= 0, 1, 2, 3…). Higher values indicate faster germination.

**Table 2.** Two-way ANOVA of final germination percentage (FGP), germination rate (GR) of seven species in relation to temperature (T) and photon irradiance (PI).

Source	*df*	FGP			GR		
		Mean Square	*F*	*P*	Mean Square	*F*	*P*
Species (S)	6	3939.6	84.3	<0.001	4960.0	650.2	<0.001
T	3	1725.7	36.9	<0.001	603.2	79.1	<0.001
S × T	18	255.7	5.5	<0.001	155.7	20.4	<0.001
Error	112	46.7			7.6		
S	6	6812.3	305.4	<0.001	2324.1	350.8	<0.001
PI	4	23056.5	1033.5	<0.001	58.0	8.751	<0.001
S × PI	24	745.2	33.4	<0.001	1575.3	237.8	<0.001
Error	140	22.3			6.6		

Data were subjected to analysis of variance (ANOVA). Before conducting ANOVA, the data were tested for homogeneity of variance, and then transformed with the arcsine square root if necessary. If the ANOVA showed significant differences, Tukey’s test was used to determine differences among treatments. All statistical analyses were performed using the SPSS 13.0 (SPSS 2004).

## Results

### Effects of temperature on germination

The effects of species, temperature and their interactions were significant for the FGP (Table 2). In general, seeds of all tested species germinated well at day/night temperatures of 25/15 °C and 30/20 °C, but they showed lower FGPs at 35/25 °C (Table 3). For *A. cristatum*, *Artemisia* *halodendron*, *C. korshinskii* and *Melilotus* *suaveolens*, seed germination was significantly inhibited under the highest day/night temperatures.

**Table 3.** Effect of day/night temperature on final germination percentage (mean ± SE, *n* = 5) of seven desert species. Different lowercase letters indicate significant differences under different temperatures for the same species. Different uppercase letters indicate significant differences among different species under the same temperature. *P *< 0.05.

Species	20/10 °C	25/15 °C	30/20 °C	35/25 °C
*A. cristatum*	72.8 ± 2.3 ABCa	67.2 ± 2.3 Ba	66.4 ± 2.0 BCa	52 ± 3.6 Bb
*Artemisia halodendron*	85.6 ± 1.0 Aa	88 ± 3.3 Aa	79.2 ± 2.7 Aa	62.4 ± 3.5 Bb
*E. dahuricus*	84.8 ± 2.7 ABab	88.8 ± 0.8 Aa	80 ± 2.2 Ab	80 ± 2.2 Ab
*C. korshinskii*	60.8 ± 4.6 Ca	55.2 ± 2.3 Aa	55.2 ± 2.9 CDa	36.8 ± 2.9 Cb
*C. microphylla*	60 ± 6.1 Ca	52 ± 3.3 Ca	63.2 ± 4.6 Ca	54.4 ± 2.4 Ba
*M. sativa*	80.8 ± 2.9 ABa	86.4 ± 1.6 Aa	78.4 ± 1.6 ABa	86 ± 2.3 Aa
*Melilotus suaveolens*	68.8 ± 3.9 BCa	62.4 ± 3.5 BCa	48 ± 2.8 Db	30.4 ± 3.2 Cc

The effects of species, temperature and their interactions were significant for the final GR (Table 2). The GRs of all tested species except for *C. microphylla* increased as the day/night temperatures increased, but they decreased under the highest day/night temperatures of 25/35 °C (Table 4).

**Table 4.** Effect of day/night temperatures on germination rate (GR) (mean ± SE, *n* = 5) of seven desert species. The significance notes for alphabets cited are same as Table 3. *P *< 0.05.

GR	20/10 °C	25/15 °C	30/20 °C	35/25 °C
*A. cristatum*	10.1 ± 0.2 Dbc	11.4 ± 0.4 Eb	12.3 ± 0.3 Ea	9.5 ± 0.6 Fc
*Artemisia halodendron*	13.4 ± 0.7 CDb	18.6 ± 0.6 Da	19.3 ± 0.6 DEa	13.4 ± 0.9 EFb
*E. dahuricus*	16.9 ± 0.5 BCc	23.3 ± 0.3 Ca	24.4 ± 0.7 CDa	20.8 ± 0.4 CDb
*C. korshinskii*	14.8 ± 1.0 BCc	20.3 ± 0.7 CDbc	25.6 ± 1.8 CDa	18.0 ± 1.7 DEbc
*C. microphylla*	17.2 ± 1.1 Bb	19.9 ± 0.9 CDb	29.5 ± 2.6 Ca	30.5 ± 2.0 Ba
*M. sativa*	41.7 ± 1.0 Ac	69.9 ± 1.1 Aa	58.7 ± 1.7 Ab	57.3 ± 1.7 Ab
*Melilotus suaveolens*	38.2 ± 0.7 Ab	43.9 ± 1.0 Bab	44.9 ± 2.1 Ba	25.0 ± 2.2 BCc

### Effects of photon irradiance on germination

**Figure 1.**
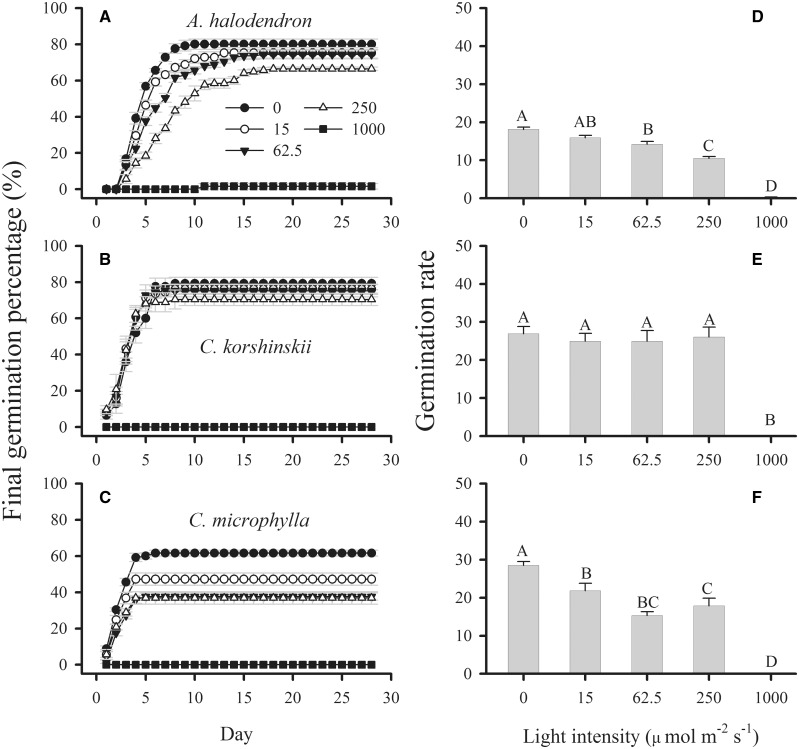
Final germination percentages (A–C) and rates (D–F) (mean ± SE, *n* = 5) of seeds of three desert shrub species under different light intensities. Different uppercase letters indicate significant differences.

The effects of species, photon irradiance and their interactions were significant for FGP (Table 2). In general, seeds showed the highest FGPs under dark conditions, and the FGP decreased as the light intensity increased (Fig. 1). For all seven species, seed germination was strongly inhibited under the highest photon irradiance (1000 µmol m^−^^2^ s^−^^1^). For all of the tested species except *C. microphylla* and *Melilotus* *suaveolens*, the FGPs under 0, 15 and 62.5 µmol m^−^^2^ s^−^^1^ photon irradiances were not significantly different. The seed germination of *C. microphylla* and *Melilotus* *suaveolens* was sensitive to light, and differed significantly among the 0, 15 and 62.5 µmol m^−^^2^ s^−^^1^ photon irradiance treatments.

Based on the seed germination responses to photon irradiance, the seven species could be divided into three groups. The first group consisted of *A. cristatum*, *Artemisia* *halodendron*, *E. dahuricus* and *C. korshinskii*. In this group, seed germination was favoured under lower photon irradiances (range from 0 to 64.5 µmol m^−^^2^ s^−^^1^) but was inhibited under high photon irradiance (1000 µmol m^−^^2^ s^−^^1^).

*Medicago* *sativa* belonged to the second group, and showed light-insensitive seed germination. Under day/night temperatures of 25/15 °C, seeds germinated well under all five light intensities. The FGP was 71.2 ± 1.5 % under the highest photon irradiance (1000 µmol m^−^^2^ s^−^^1^).

*Caragana* *microphylla* and *Melilotus* *suaveolens* belonged to the third group. Seed germination of these two species significantly decreased as the photon irradiance increased from 0 to 15 µmol m^−^^2^ s^−^^1^ for *C. microphylla* and from 15 to 62.5 µmol m^−^^2^ s^−^^1^ for *Melilotus* *suaveolens*.

**Figure 2.**
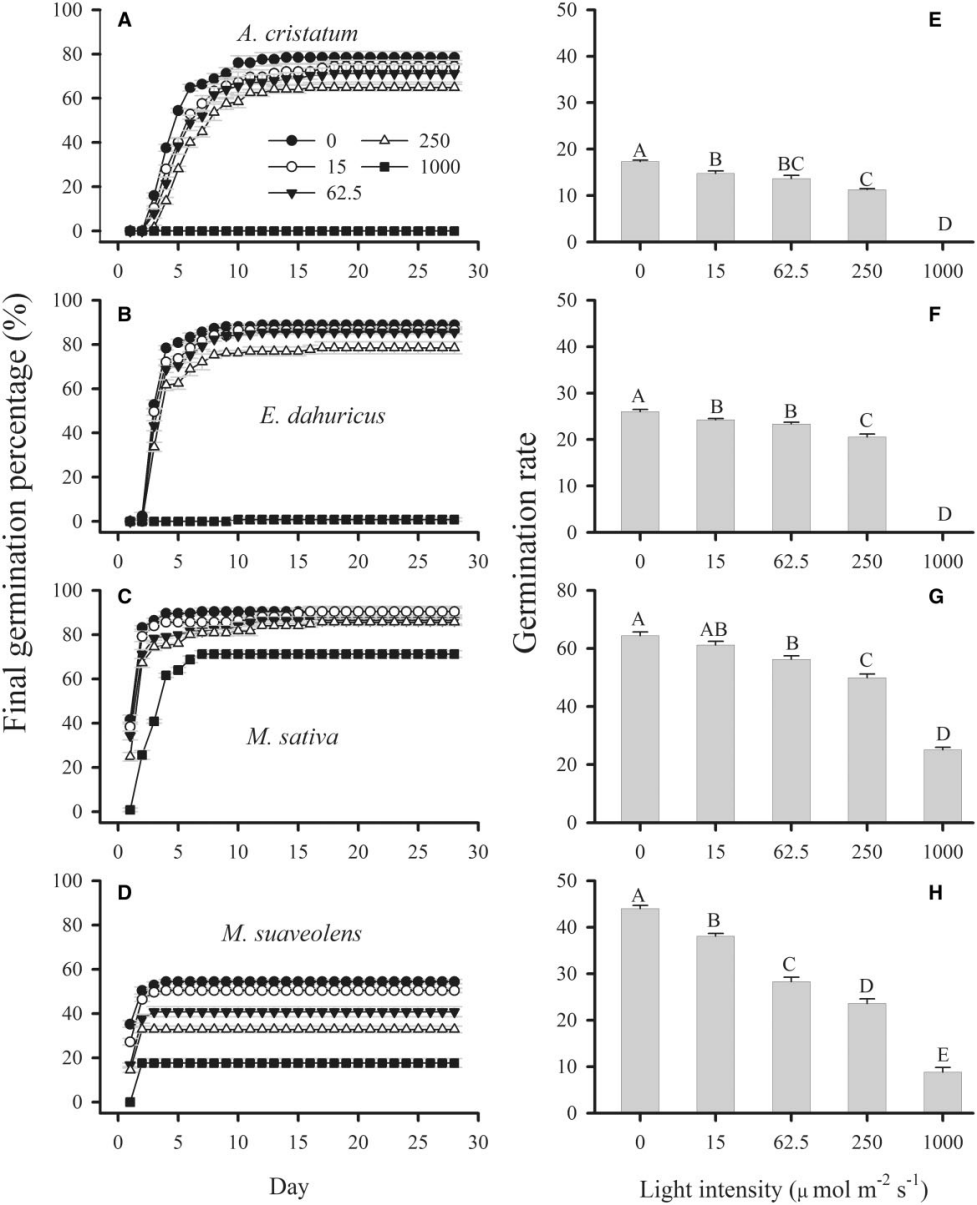
Final germination percentages (A–D) and rates (E–H) (mean ± SE, *n *= 5) of seeds of four desert shrub species under different light intensities. Different uppercase letters indicate significant differences.

The GR also varied significantly with photon irradiance, species and their interaction (Table 2). Seeds of all seven species germinated fastest in the dark, and the GR decreased as the photon irradiance increased (Figs 1 and 2). Among the seven species, *M. sativa* showed the highest GR under all five photon irradiances; *A. cristatum* and *Artemisia* *halodendron* had the lowest GRs. For most species, the germination percentage stabilized after 5 days in the dark, but took longer to stabilize under higher photon irradiance.

## Discussion

During the life of a plant, seeds show the greatest tolerance to environmental stresses, but seedlings are the most sensitive (Gutterman 1993). Thus, the successful establishment of vegetation strongly depends on the adaptation of seed germination to environmental factors. The environmental control of seed germination is a complex process, and seeds can only germinate when environmental stresses do not exceed their limits of tolerance (Baskin and Baskin 2014). Studies on the adaptation of plants, especially during germination, are useful for selecting appropriate species for vegetation restoration.

### Effects of temperature on seed germination

Germination cues reflect the climate conditions under which the species is most likely to succeed in recruitment (Schütz and Rave 1999). In the field, temperature can effectively induce or relieve seed dormancy, and thus it inhibits seed germination under unfavourable conditions (Adondakis and Venable 2004).

Temperature requirements for seed germination vary among species (Baskin and Baskin 2014). As temperature requirements for germination are closely related to germination time, varieties of species distributed in different regions have their own temperature requirements for germination. Typical desert plant species can germinate within a wide temperature range of 5–40 °C (Gutterman 1993). In our study, the suitable day/night temperatures for germination of *A. cristatum*, *Artemisia* *halodendron*, *C. korshinskii*, *C. microphylla* and *Melilotus* *suaveolens* ranged from 20/10 °C to 30/20 °C. For *E. dahuricus* and *M. sativa*, the FGPs exceeded 80 %, indicating that these two species have a wide optimal temperature ranges and that temperature is not the critical factor limiting seed germination in the field.

Previous reports have stated that the lower temperature limit is related to ecological adaptation (Grime *et al.* 1981), while the upper limit is caused by physiological constraints (Hilhorst 1993). The results of our study illustrated that the FGPs showed different patterns under the four temperature regimes.

Based on the results of our study, the seven species formed three groups based on their germination responses to temperature. The first group, consisting of *E. dahuricus*, *C. microphylla* and *M. sativa*, showed temperature-independent seed germination. The second group (*A. cristatum*, *Artemisia* *halodendron* and *C. korshinskii*) showed significantly inhibited seed germination under the highest day/night temperatures (35 /25 °C) but no significant difference in seed germination among the other temperature regimes. The third group (*Melilotus* *suaveolens*) showed inhibited seed germination under higher temperatures and stimulated seed germination under lower temperatures.

For *A. cristatum*, *Artemisia* *halodendron*, *C. korshinskii* and *Melilotus* *suaveolens*, germination was significantly inhibited under the highest day/night temperatures. In the Horqin Sandy Land, the mean temperature on the soil surface is 12.3 °C in April and 20.8 °C in May (Zhang *et al.* 2005). These temperatures are suitable for seed germination of species in Groups 2 and 3, including *A. cristatum*, *Artemisia* *halodendron*, *C. korshinskii* and *Melilotus* *suaveolens*, but higher temperatures significantly inhibited their germination. Because most precipitation in the Horqin Sandy Land occurs from May to September, this period would be suitable for seedling establishment. Thus, seeds of these four species should be sown from April to May.

### Effects of photon irradiance on seed germination

The light responses of seeds are important to ensure that they germinate at appropriate places and times that are favourable for seedling establishment (Baskin and Baskin 2014). Seeds dispersed onto the soil surface may be subjected to strong photon irradiances. In sandy areas, seeds are buried at different depths depending on prevailing wind speeds and other habitat characteristics (Maun 1998). The burial of seeds can significantly and rapidly decrease photon irradiance, according to our measurements (Lai *et al.* 2015). An understanding of the different light requirements for seed germination can increase the chances of successful establishment in sandy areas.

Seeds that settle on the surface of sand are exposed to strong light, high temperatures, high evaporation rates and low moisture. All of these factors can inhibit seedling establishment. Thus, a dark requirement for germination would be adaptive for species distributed in sandy areas. Several studies have shown that the germination of many plants that inhabit sandy soils were stimulated by dark conditions (Gutterman 1993; Zheng *et al.* 2004, 2005*a*,*b*; Lai *et al.* 2010). In our study, the seeds of all seven species germinated well in the dark, indicating that burial in sand can increase the germination of these species. Although burial in sand may result in favourable conditions for seed germination, the shoot may not be able to reach the surface. Thus, species with small seeds (weighing <0.1 mg) often require light for germination (Grime *et al.* 1981). In the present study, higher light intensities did not increase the FGPs for all seven species, possibly because their seeds were not small enough to be considered as ‘small seeds’, as reported by Grime *et al.* (1981).

The inhibitory effect of strong photon irradiance on seed germination has been demonstrated for many species, even those that are positively photoblastic (Pons 2000). In our study, the seeds of five species (*A. cristatum*, *Artemisia* *halodendron*, *E. dahuricus*, *C. korshinskii* and *C. microphylla*) could not germinate under the highest photon irradiance (1000 µmol m^−^^2^ s^−^^1^). For the other two species, seeds were able to germinate under the highest photon irradiance, especially those of *M. sativa* (FGP > 70 %). Under natural conditions, *Melilotus* *suaveolens* and *M. sativa* are mainly distributed on fixed sand dunes, and light did not trigger their seed germination as it did for the other five species.

Sensitivity to strong light provides a mechanism to reduce the probability of seedling death on the soil surface. Seeds of some species are sensitive to light and germinate only under low-light conditions (Baskin and Baskin 2014). In this study, we observed that the FGPs of *C. microphylla* were significantly decreased at low photon irradiance (Fig. 1). This can be partly explained by the distribution of this species on semi-fixed and fixed sand dunes. In a previous study, we found that the highest seedling emergence percentage of *C. microphylla* was at a burial depth of 1.5-cm in sand, where photon irradiance was almost zero (Lai *et al.* 2015). Thus, the seed germination characteristics of *C. microphylla* under different photon irradiances are a useful adaptation to its environment.

In arid and semiarid environments, there are long intermittent drought periods that are fatal to rapid germinators. Rapid germinators germinate at the first rain event, but slow germinators require a long wetting period to germinate (Gutterman 1993). Among the seven species, *M. sativa* and *Artemisia* *halodendron* had the fastest and the slowest GR, respectively. The slow GR strategy of *Artemisia* *halodendron* could prevent seeds from germinating after one rain event that might follow a long drought period, and indicated that it was well-adapted to drought. In the Horqin Sandy Land, *Artemisia* *halodendron* is the dominant species, which reflects its strong adaptation to this arid region.

### Selections of the seven species for vegetation restoration

There are many reports about applications of the seven species: *A. cristatum* is considered as biological invasion species in the ecological restoration process (Ambrose and Wilson 2003; Bakker and Wilson 2004); *Artemisia* *halodendron* is regarded as an indicator plant for the semi-fixed sandy land (Li and Zhang 1991); *C. korshinskii*, *C. microphylla* and *M. sativa* are selected as air seeding species in Mu Us sandy land (Zheng *et al.* 2003), *M. sativa* and *E. dahuricus* are drought tolerant forage in sandy region and widely used in restoration of abandoned fields on the Loess Plateau, China (Li and Zhang 1991; Li *et al.* 2008). However, there are no specific suggestions about selections of these seven species in the sandy land.

The Horqin Sandy Land has a complex landscape that includes shifting, semi-fixed, and fixed sand dunes (Su and Zhao 2003). The vegetation succession of this area can be summarized as: (i) colonization of pioneer species on bare shifting sand dunes; (ii) establishment of secondary species on semi-fixed sand dunes and (iii) establishment of climax species on fixed dunes (Liu 1985). All of the seven studied species germinated well in the dark, indicating that sand burial can increase their seed germination. The shifting sand dunes habitat may be fatal to rapid germinators, but slower germinators could show a cumulative germination pattern over an extended period, even if it is interrupted by periods of drought (Fenner and Thompson 2005). Among the seven species studied here, *Artemisia* *halodendron* and *A. cristatum* are the best adapted to shifting sand dunes, and were found to establish after *Agriophyllum squarrosum* in a field survey (Liu 1985). On the semi-fixed sand dunes, the soil surface is covered by sparse vegetation, and the soil moisture content is higher than that in shifting sand dunes. Thus, *E. dahuricus*, *C. korshinskii* and *C. microphylla* could be selected for restoration of shifting sand dunes due to their higher seed germination percentages in the dark, and their relatively slow germination rates. The seeds of both *M. sativa* and *Melilotus* *suaveolens* germinated rapidly, a characteristic that requires a higher soil moisture content for seedling survival. In fact, these two species are artificially cultivated as pasture, and so both of them would be suitable for revegetation of fixed sand dunes. After long-term evolutionary adaptation to its environment, *M. sativa* may have evolved different seed germination behaviour compared with those of the other studied species, because its seeds germinated well under all photon irradiances, even high photon irradiance.

The timing of seed dispersal is an important factor for vegetation restoration. The seeds of all seven species germinated best in the dark. Therefore, burial in sand is important for their germination in the field. According to the local weather data, the mean wind velocity in April was 4.46 m/s, and most of the windy days were in March, April and May. The precipitation in May was 35.72 mm, and the soil temperature at the surface and at 5-cm soil-depth were 20.83 and 18.00 °C, respectively (Zhang *et al.* 2005; Cui *et al.* 2007). Thus, seeds distributed/sown in early May would have a greater chance of being buried in sand, and the precipitation and temperature conditions in May would be suitable for seedling survival.

In the study, we evaluated the seed germination responses of seven key plant species of desertified areas, and explored the implications for vegetation restoration. Both temperature and photon irradiance showed great effects on seed germination of these species. The seeds germinated well at day/night temperatures of 25/15 °C and 30/20 °C in the dark. During applications of the seven species in the restoration project, seed germination characteristics should be considered at specific landscape.

## Sources of Funding

This work was funded by the National Natural Science Foundation of China (Grant Nos 41330749 and 41401105).

## Contributions by the Authors

L.C., Y.Z. and H.S. conceived and designed the experiments. L.C. and J.Z. performed the experiments. L.L., L.J. and Y.Z. analyzed the data. L.L. and Y.Z. wrote the manuscript.

## Conflicts of Interest Statement

None declared.
